# Novel biallelic mutations in *TMEM126B* cause splicing defects and lead to Leigh-like syndrome with severe complex I deficiency

**DOI:** 10.1038/s10038-022-01102-4

**Published:** 2022-12-08

**Authors:** Xiyue Zhou, Xiaoting Lou, Yuwei Zhou, Yaojun Xie, Xinyu Han, Qiyu Dong, Xiaojie Ying, Mahlatsi Refiloe Laurentinah, Luyi Zhang, Zhehui Chen, Dongxiao Li, Hezhi Fang, Jianxin Lyu, Yanling Yang, Ya Wang

**Affiliations:** 1 Key Laboratory of Laboratory Medicine, Ministry of Education, Zhejiang Provincial Key Laboratory of Medical Genetics, School of Laboratory Medicine and Life sciences, Wenzhou Medical University, Wenzhou, 325035 Zhejiang China; 2 Center for Reproductive Medicine, Department of Genetic and Genomic Medicine, Zhejiang Provincial People’s Hospital, Affiliated People’s Hospital, Hangzhou Medical College, Hangzhou, Zhejiang 310014 China; 3 Department of Pediatrics, Peking University First Hospital, 100034 Beijing, China; 4 Laboratory Medicine Center, Department of Clinical Laboratory, Zhejiang Provincial People’s Hospital, Affiliated People’s Hospital, Hangzhou Medical College, Hangzhou, Zhejiang 310053 China

**Keywords:** Disease genetics, Neurodegeneration

## Abstract

Leigh syndrome (LS)/Leigh-like syndrome (LLS) is one of the most common mitochondrial disease subtypes, caused by mutations in either the nuclear or mitochondrial genomes. Here, we identified a novel intronic mutation (c.82-2 A > G) and a novel exonic insertion mutation (c.290dupT) in *TMEM126B* from a Chinese patient with clinical manifestations of LLS. In silico predictions, minigene splicing assays and patients’ RNA analyses determined that the c.82-2 A > G mutation resulted in complete exon 2 skipping, and the c.290dupT mutation provoked partial and complete exon 3 skipping, leading to translational frameshifts and premature termination. Functional analysis revealed the impaired mitochondrial function in patient-derived lymphocytes due to severe complex I content and assembly defect. Altogether, this is the first report of LLS in a patient carrying mutations in *TMEM126B*. Our data uncovers the functional effect and the molecular mechanism of the pathogenic variants c.82-2 A > G and c.290dupT, which expands the gene mutation spectrum of LLS and clinical spectrum caused by *TMEM126B* mutations, and thus help to clinical diagnosis of *TMEM126B* mutation‐related mitochondrial diseases.

## Introduction

Leigh syndrome (LS, MIM 256000) is the most prevalent childhood-onset mitochondrial disease, with neurodegenerative changes as its most distinctive feature [1], which has a prevalence of approximately 1:40,000 and is highly genetically heterogeneous, primarily with impaired mitochondrial energy production [2]. LS exhibits several different modes of inheritance (X-linked, autosomal or maternal). To date, more than 75 disease-causing genes including both mitochondrial and nuclear genes have been identified [3]. Although defects in each of the five oxidative phosphorylation system (OXPHOS) complexes have been observed in LS patients [4], nearly one-third of LS cases are related to complex I deficiency. Mitochondrial complex I is located in the first step of the mitochondrial oxidative respiratory chain, which contributes ~40% of the electrochemical proton gradient to make ATP from ADP and pump the electron to generate the mitochondrial membrane potential [5, 6]. In addition to bioenergetic role, the complex I transfers electron from NADH to form NAD^+^ and drives the single electrons to oxygen to form superoxide, thus promoting tricarboxylic acid (TCA) cycle and fatty acid oxidation. Isolated complex I deficiency causes a wide range of metabolic/bioenergetics related diseases, including Leigh syndrome, Leigh-like encephalomyopathy, Leber’s hereditary optic neuropathy (LHON, MIM 535000), mitochondrial myopathy, encephalomyopathy, lactic acidosis and stroke-like episodes (MELAS, MIM 540000), nonsyndromic hearing loss, cardiomyopathy and myopathy [7]. The phenotypes are highly heterogeneous but mainly present in the nervous system and skeletal muscles [8]. LS is characterized by the degeneration of the central nervous system (brain, spinal cord, optic nerve, etc.), which manifested with loss of motor skills, vomiting, irritability or seizure, hypotonia and lactic acidosis, and finally impaired respiratory and kidney function. In addition to typical LS, some respiratory chain deficiencies manifested with multiple system disorders such as peripheral nerves, heart, liver, kidney, etc., which identified as Leigh-like syndrome (LLS) [9].

*TMEM126B* (MIM 615533) contains five exons and encodes a component of mitochondrial complex I intermediate assembly (MCIA) complex, which is required for assembly of complex I but is not part of the mature complex [10]. Mutations in *TMEM126B* would cause an isolated mitochondrial complex I deficiency and result in various clinical phenotypes such as exercise intolerance, muscle weakness, hyperlactic acidemia, hypertrophic cardiomyopathy and renal tubular acidosis [11–13]. To date, ten patients with a total of four mutations in *TMEM126B* have been reported worldwide, but all patients reported had a normal neurological presentation [11–13]. Notably, the genetic spectrum of the *TMEM126B* mutations in China remains unclear.

In this study, using next-generation sequencing in a Chinese patient manifested with LLS, we identified two novel heterozygous mutations of *TMEM126B* (c.82-2 A > G and c.290dupT). Bioinformatics analysis and functional assays revealed that c.82-2 A > G mutation caused complete exon 2 skipping and c.290dupT induced partial and complete exon 3 skipping. Patient-derived lymphoblastoid cells carrying biallelic mutations exhibited complex I content and assembly defects and mitochondrial dysfunction. Our findings uncovered the functional effect and the molecular mechanism of the pathogenic *TMEM126B* variants c.82-2 A > G and c.290dupT, which not only expand the gene mutation spectrum of LLS, but also expand the clinical spectrum caused by *TMEM126B* mutations, thus contributing to the clinical diagnosis of *TMEM126B* mutation-related mitochondrial diseases.

## Material and methods

### Patient and ethics approval

The patient was admitted to the Department of Pediatrics at Peking University First Hospital and the clinical diagnosis of LLS was established in accordance with previously published criteria [14]. The study received ethical review from Peking University First Hospital (2017–217). The written informed consent were obtained from the participant’s legal guardian and family members.

### Variants analysis

Whole exome sequencing (WES) and mitochondrial genomic sequencing were conducted using Illumina HiSeq 2000 sequencer (Illumina, USA). In brief, DNA was isolated from patient peripheral blood and the quality control was checked using agarose gel electrophoresis. DNA libraries were prepared comprises end-repair of fragmented DNA, A-tailing, adapter ligation and amplification. After array capturing, sequencing was performed on the Illumina HiSeq 2000 sequencer (Illumina, USA) platform with paired-end 200-bp reads and the average coverage rate more than 20 ×. Poor-quality sequence data and adapter sequence were removed to control the quality of the raw data. Then the quality-controlled sequencing reads were mapped to the reference sequence (GRCh38/hg38), and base quality score recalibration was performed using base recalibrator and print reads. Germline short variants (SNPs, insertions or deletions) were then detected by the genome analysis toolkit (GATK). The candidate disease-causing variants were prioritized as follows: (a) variants should conform to Mendelian laws of inheritance; (b) allele frequency should be less than 1% in the population databases, such as ExAC (http://exac.broadinstitute.org/); (c) the variant should be null variant (nonsense, frameshift, canonical splice sites, initiation codon, single or multiexon deletion in highly conserved region); (d) in silico prediction should be pathogenic or likely pathogenic; (e) correlation between genotype and phenotype. For variants validation, the recessive segregation study in *TMEM126B* variants were confirmed using sanger sequencing.

### Bioinformatics analyses of variants

The population allele frequency and pathogenicity of both variants were predicted by the following online bioinformatics analysis tools: 1000 genomes (https://www.internationalgenome.org/) [15], gnomAD (https://gnomad.broadinstitute.org/) [16], ExAc (http://exac.broadinstitute.org) [17], dbSNP (https://www.ncbi.nlm.nih.gov/snp/) [18], Clinvar (https://www.ncbi.nlm.nih.gov/clinvar/) [19] and HGMD (Human Gene Mutation Database, http://www.hgmd.cf.ac.uk/ac/index.php) [20]. Pathogenicity of both variants was analyzed according to the American College of Medical Genetics and Genomics guidelines [21]. The effect of two variants on *TMEM126B* mRNA splicing were predicted by using alternative splice site predictor (ASSP) (http://wangcomputing.com/assp/) [22] and exonic splicing enhancers (ESE) finder (http://krainer01.cshl.edu/tools/ESE2/) [23].

### Minigene construction and expression

The minigene expression plasmids were constructed as previously reported [24] and in vitro splicing confirmation was performed as aforementioned [25]. Briefly, the vector was cleaved with *Bam*HI (Takara Bio, USA) and *Eco*RI (Takara Bio), and then ligated the DNA segment containing the two variant sites to a cloning expression plasmid, pSPL3, using T4 ligase (NEB, USA) following the manufacturer’s instruction. The primers were listed in Supplement Table 1. The minigene plasmid was transfected into HEK293T cells (Cell Bank of the Chinese Academy of Science) with lipofectamine 3000 reagent (Thermo Fisher Scientific, USA) based on manufacturer’s instruction.

### Cell culture

Immortalized lymphocytes derived from II-1 and healthy controls were conducted using epstein-barr virus procured from B95-8 cells (Cell Bank of the Chinese Academy of Science, China) as mentioned before [26]. Immortalized B lymphocytes were culture in Roswell Park Memorial Institute (RPMI) 1640 medium (Sigma-Aldrich, USA) supplement with 10% fetal bovine serum (Sigma-Aldrich), 1% (v/v) penicillin-streptomycin (Beyotime Biotechnology) and 0.25 μg/mL amphotericin B (Beyotime Biotechnology). HEK293T cells were cultured in Dulbecco’s Modified Eagle Medium (DMEM) medium (Sigma-Aldrich) supplement with 12% fetal calf serum (Sigma-Aldrich) and 1% (v/v) penicillin-streptomycin and 0.25 μg/mL amphotericin B. All cells were cultured in 37 °C incubator with 5% CO_2_.

### Reverse transcriptase PCR (RT-PCR) analysis

After transfecting the minigene plasmid into HEK293T for 48 h, the cells were collected and then performed RT-PCR that amplify the fragment containing the mutant site to evaluate the mRNA splicing. Briefly, total RNA was extracted using TRIzol reagent (Sigma-Aldrich) and then reverse transcribed to cDNA by using reverse transcription kit (Vazyme) according to the instruction. Agarose gel electrophoresis (BioFroxx, Germany) was used to separate the segment of PCR products and the sequence of each DNA product was determined by Sanger sequence. All primers were listed in Supplementary Table 1.

### Western blot

Western blot was conducted as before [27]. In brief, cells were washed with pre-cold PBS and lysed in lysis buffer (Cell signaling technology, USA) supplemented with 1 mM PMSF (Sigma-Aldrich). Samples were measured the concentration using Pierce BCA Protein Assay kit (Thermo Fisher Scientific) followed by centrifugation at 14,000 × *g* for 10 min at 4 °C. The protein lysate was denatured with loading buffer for 5 min at 95 °C and loaded onto 10% bis-tris protein gels. Proteins were transferred onto immune-blot polyvinylidene difluoride membrane (BIO-RAD, USA) and blocked with 5% w/v milk. The membranes were incubated with second HRP-conjugated secondary antibodies for 2 h followed by primary antibodies overnight. The protein signals were visualized using SuperSignal West Pico Chemiluminescent Substrate (BIO-RAD) and quantified grayscale measurements in Gel-Pro analyzer 4.0 (USA). The antibodies are as follows: TMEM126B (Abmart, China, 1:1000), β-actin (Santa cruz, USA, 1:2000), Anti-mouse IgG, HRP (Cell Signaling technology, 1:2000) and Anti-rabbit IgG, HRP (Cell Signaling technology, 1:2000).

### Blue native polyacrylamide gel electrophoresis

Blue native polyacrylamide gel electrophoresis (BN-PAGE) was conducted as mentioned before [27]. For mitochondrial supercomplexes, 1% digitonin (Sigma-Aldrich) was used to dissolve the membrane protein from patient and controls, then 3.5–16% gradients polyacrylamide gel to separate the supercomplexes. For mitochondrial complexes, 1% dodecyl maltoside (DDM, Sigma-Aldrich) was used and then 3–11% gradient polyacrylamide gel to separate the complexes. The proteins were transferred on the PVDF membrane (Bio-Rad, USA), blocked with 5% no-fatty acid milk (Mengniu, China), incubated with the first and second antibodies, and then detected the protein signals using. The following antibodies were used: anti-Grim19 (1:1000, Sigma-Aldrich), anti-SDHA (1:3000, Sigma-Aldrich), anti-UQCRC2 (1:2000, Sigma-Aldrich), anti-MT-COI (1:2000, Sigma-Aldrich), anti-ATP5A (1:3000, Sigma-Aldrich), anti-NDUFB6 (1:2000, Sigma-Aldrich), anti-NDUFS3 (1:2000, Sigma-Aldrich), anti-TOM70 (1:2000, Proteintech, China), anti-mouse IgG HRP linked (1:2000, Cell Signaling Technology), anti-rabbit IgG HRP linked (1:2000, Cell Signaling Technology), anti-mouse IgG, AP (1:2000, Cell Signaling Technology).

### Oxygen consumption rate

Oxygen consumption rate was performed as before [27]. In short, 5 × 10^6^ immortalized lymphocytes from the candidate and the age-matched healthy controls were rapidly and gently harvested and then oxygen consumption rate was analyzed using the oxygraphy-2k detector (Oroboros, Austria). Oligomycin (0.1 mg/mL, Sigma-Aldrich) was used to measure the ATP-linked respiration and the carbonyl cyanide 4-(trifluoromethoxy) phenylhydrazone (0.1 mM, Sigma-Aldrich) was added to detect the maximal respiration and the reserve respiratory capacity.

### Cellular ATP content and mitochondrial ROS level detection

Cellular ATP and mitochondrial ROS content were measured by ATP bioluminescent assay kit (Sigma-Aldrich) and MitoSOX™ Red reagent (Thermo Fisher Scientific) following the manufacturer’s instruction, respectively [27]. For cellular ATP content measurement, immortalized lymphocytes from patient and age-matched healthy controls were collected and resuspended with filtered ultrapure water. Then added the cell suspension to the ATP-releasing solution and detected the autofluorescence. The protein concentration of the samples was measured with the BCA protein concentration assay kit (Thermo Fisher Scientific) as a calibration. For the mitochondrial ROS level detection, immortalized lymphocytes from patients and age-matched healthy controls were collected and resuspended in working solution containing 5uM MitoSOX reagent. Then incubated at 37 °C for 10 min at dark, gently washed cells and detected the fluorescence.

### Statistical analysis

All experiments were conducted at least three times independently and analyzed by mean ± SEM using GraphPad prism 8.0 (USA). *P* values were calculated using independent Student’s *t* test or one-way ANOVA, *p* < 0.05 was considered statistically significant.

## Results

### Clinical manifestation and genetics analysis

**Fig. 1:**
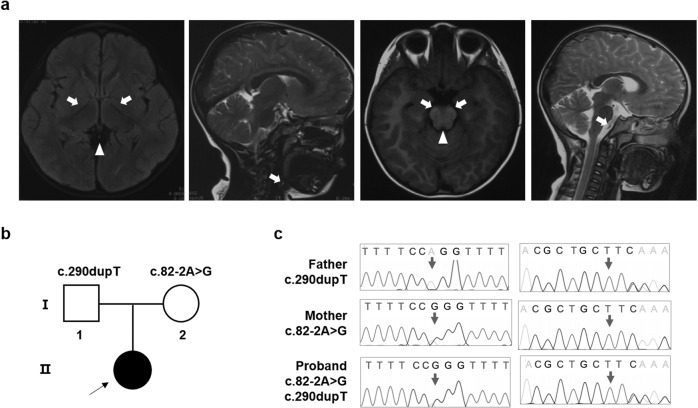
Segregation analysis, sanger sequence and MRI manifestation. **a** MRI image of proband. The arrow indicated the patchy, spot-like, long T1 and T2 signal shadows symmetrically in cerebral peduncles corticospinal tract. The triangle indicated the flakes T2WI in the cerebral bridge. **b** Segregation analysis of affected individual. Rectangles indicate males, circles female, and solid circle represents affected individual. The proband was pointed out by arrow. **c** Sanger sequence of the affected family. The arrows indicate mutation sites

The individual (II-1) was born into nonconsanguineous healthy Chinese family with a normal gestational and delivery records. She fell the development milestone, with unsteady gait at the age of 15 months, and received rehabilitation training for two months, however, no improvement in her symptoms. By age 2, she developed uninterrupted strabismus in her left eye and still showed gait disturbance. When she was three years old, the candidate walked with drunken gait, along with weak muscular strength, reduced muscle tension, malnutrition but well developed reflexes and mental response. The face of patient was yellow, with yellow palms and feet, and the back of the neck was ciliated. Magnetic resonance imaging (MRI) in the cerebral peduncles corticospinal tract demonstrated patchy, spot-like, long T1 and T2 signal shadows symmetrically and fluid attenuated inversion recovery (FLAIR) showed equal signal. MRI in the cerebral bridge appeared flakes T2 weighted image (T2WI), hypo-intense T2WI FLAIR signal and spot-like hypo-intense apparent diffusion coefficient (ADC) signal (Fig. 1a). Electromyogram/evoked potentials test (EMG/EP) revealed that sensory nerve conduction slows in the left gastrocnemius muscle (35.3 m/s, normal range 40–60 m/s) and motor nerve conduction was normal. No further sensory testing was done due to the child was unable to cooperate. Metabolic investigation showed increased blood lactate (3.14, normal range 0.50–2.20 mM) and β- hydroxybutyric acid (1.18, normal range 0.02–0.27 mM). Amino acid and acylcarnitine profile analysis were normal. Other tests showed increased aspartate amino acid transferase (42.2, normal range 0.0–35.0 U/L), lactate dehydrogenase (247.3, normal range 109.0–245 U/L) and α-hydrobutyrate dehydrogenase (260.8, normal range 90.0–250.0 U/L), indicating an impaired liver function. The symptom was partly overlap with LS (progressive neurological impaired with motor developmental delay and raised blood lactate level). However, the neurological abnormal signs was focus on cerebral peduncles corticospinal and cerebral bridge, thus this patient was diagnosis with LLS.

Next-generation sequencing was performed to detect the disease-causing gene of the probing, after the filtering with established criteria [27], a novel splice site variant (c.82-2 A > G, intron 1) and a novel insertion variant (c.290dupT, exon 3) in *TMEM126B* (NM_018480.7) were identified and no clinically significant mitochondrial genomic-related variants were detected. Segregation analysis confirmed that c.82-2 A > G was maternal inherited while c.290dupT was paternal (Fig. 1b, c).

### A comprehensive in silico analysis of c.82-2 A > G and c.290dupT

**Fig. 2:**
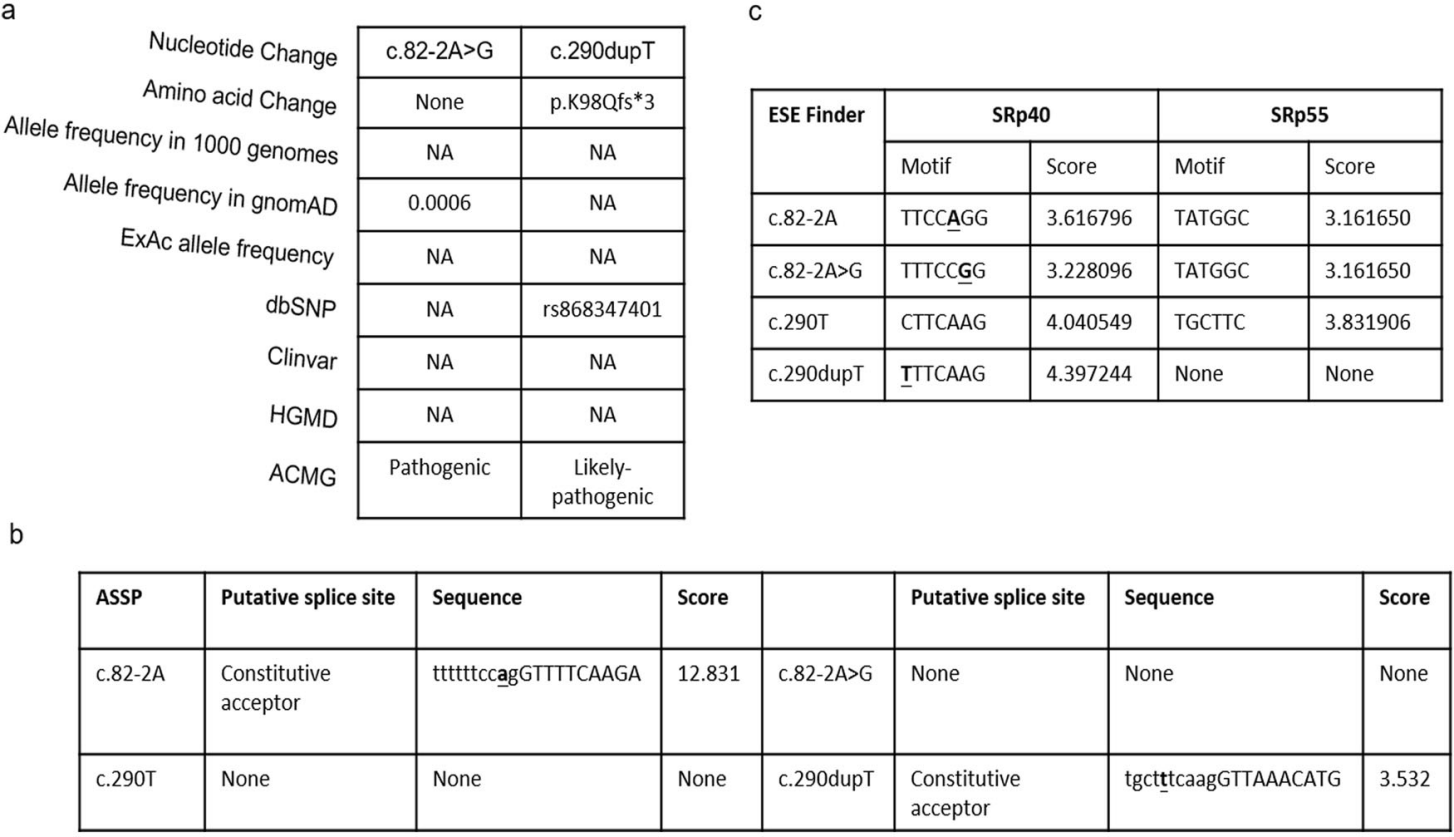
In silico analysis of *TMEM126B* variants. **a** Bioinformatic analysis of allele frequency and pathogenicity prediction about two variants. **b** Putative splice sites and scores for wild-type and mutant sites predicted from ASSP. **c** Splicing regulatory protein binding motif predicted by ESE Finder for wild-type and mutants. Mutation sites are highlighted. NA not available, HGMD Human Gene Mutation Database, ACMG the American College of Medical Genetics and Genomics

To investigate the potential pathogenicity of the above two variants (c.82-2 A > G and c.290dupT), we performed a series of bioinformatics analyses. As shown in Fig. 2a, the allele frequency of c.82-2 A > G in gnomAD was extremely low (0.0006), and neither of them had record in several population variation frequency and pathogenicity prediction databases (Fig. 2a). The two variants showed co-segregation with an autosomal recessive trait. In silico prediction showed that the c.82-2 A > G variant could cause exon 2 skipping, and that the c.290dupT cause frameshift, thus resulting in a premature stop codon. According to ACMG guideline, the c.82-2 A > G variant was predicted as pathogenic and c.209dupT was likely-pathogenic (Fig. 2a). ASSP (Alternative Splice Site Predictor) indicates that the c.82-2 A > G mutation may destroy original splice constitutive acceptor, while the frameshift mutation c.290dupT may create a new splice constitutive acceptor (Fig. 2b). ESE finder predicted that the c.290dupT variant would loss a putative SRp55 binding site (Fig. 2c). Taken together, these results indicate that both c.82-2 A > G and c.290dupT variants might disease-causing and affect pre-mRNA splicing.

### Identification of variants affecting *TMEM126B* splicing by using a minigene splicing assay

**Fig. 3:**
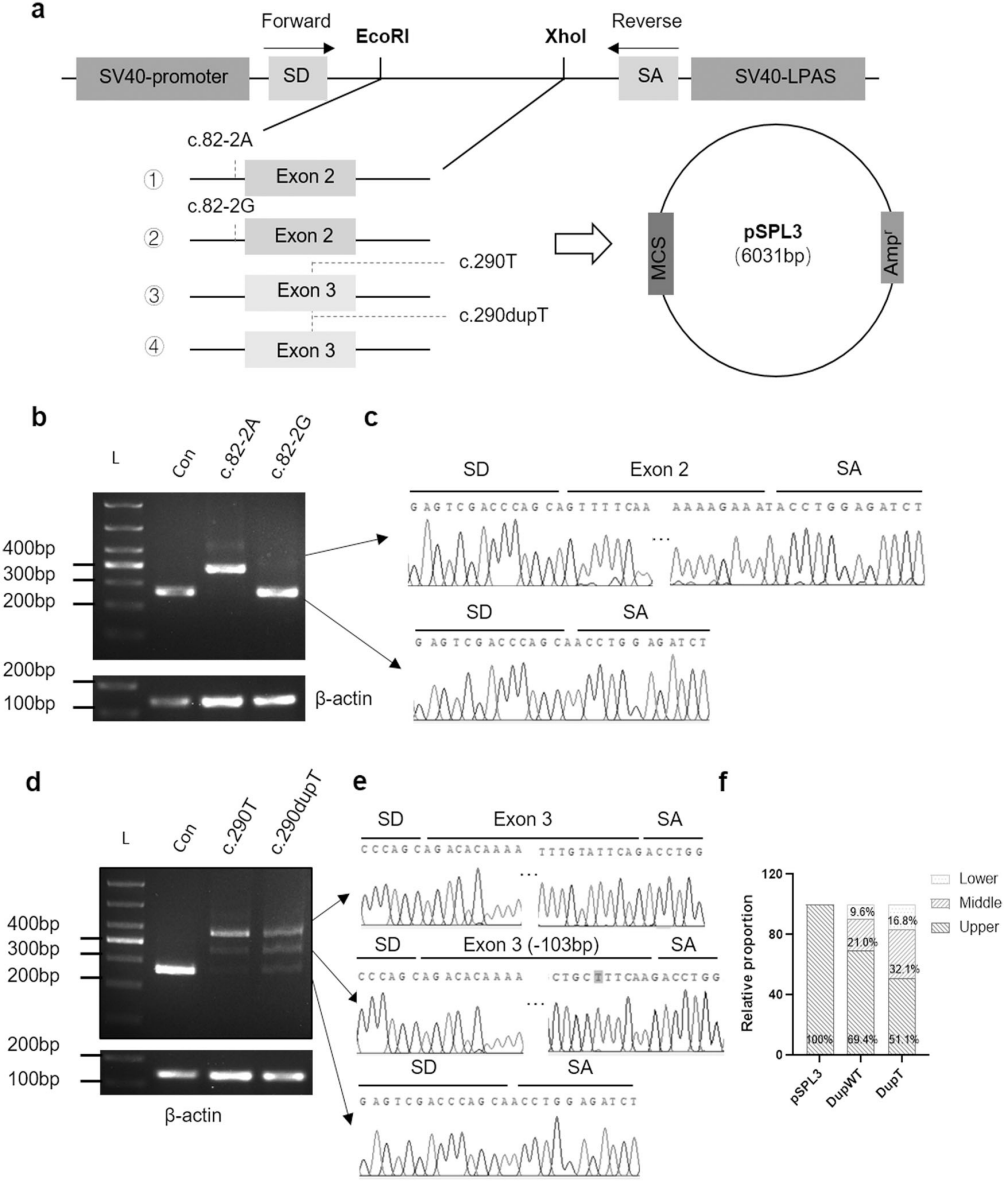
The minigene splicing assays based on the pSPL3 exon trapping vector. **a** Schematic diagram of the in vitro minigene plasmid construction. The pSPL3 plasmid contains two exons, SD and SA. The primers were specify to exon SD and SA, respectively. Agarose gel electrophoresis (**b**) and Sanger sequencing (**c**) for the PCR products of c.82-2 A and c.82-2 A > G mutant. β-actin was used as loading control. Agarose gel electrophoresis (**d**) and Sanger sequencing (**e**) for the PCR products of c.290 T and c.290dupT mutant. The c.290dupT was highlight with gray background. **f** Relative quantification of each segment of PCR product from pSPL3 control, c.290 T and c.290dupT

To explore whether the c.82-2 A > G and c.290dupT variants influence mRNA splicing, we conducted an exon trapping assay based on pSPL3 plasmids (Fig. 3a). RT-PCR and Sanger sequencing showed that both the empty pSPL3 control and c.82-2 G mutant constructs gave rise to a 263-bp PCR fragment missing exon 2 of *TMEM126B* gene, whereas the wild-type c.82-2 A yielded a RT-PCR product of 385-bp containing exon 2 (Fig. 3b, c), which indicated that c.82-2 A > G mutation destroyed the original splice acceptor site and resulted in full exon 2 skipping. The plasmid constructs of both wild-type c.290 T and mutant c.290dupT expressed three transcripts, including a transcript without exon 3, a transcript with 103-bp deletion of exon 3 and a transcript containing exon 3 but with one base (T) duplication (Fig. 3d, e). Quantitative analysis showed that natural partial and complete exon 3 skipping were weak in wild-type c.290 T construct, but significantly increased in mutant c.290dupT (Fig. 3f). Altogether, our data suggested that c.82-2 A > G mutation caused complete exon 2 skipping and c.290dupT induced partial and complete exon 3 skipping.

### Confirmation of variant-induced spliceogenicity in patient-derived lymphocytes

**Fig. 4:**
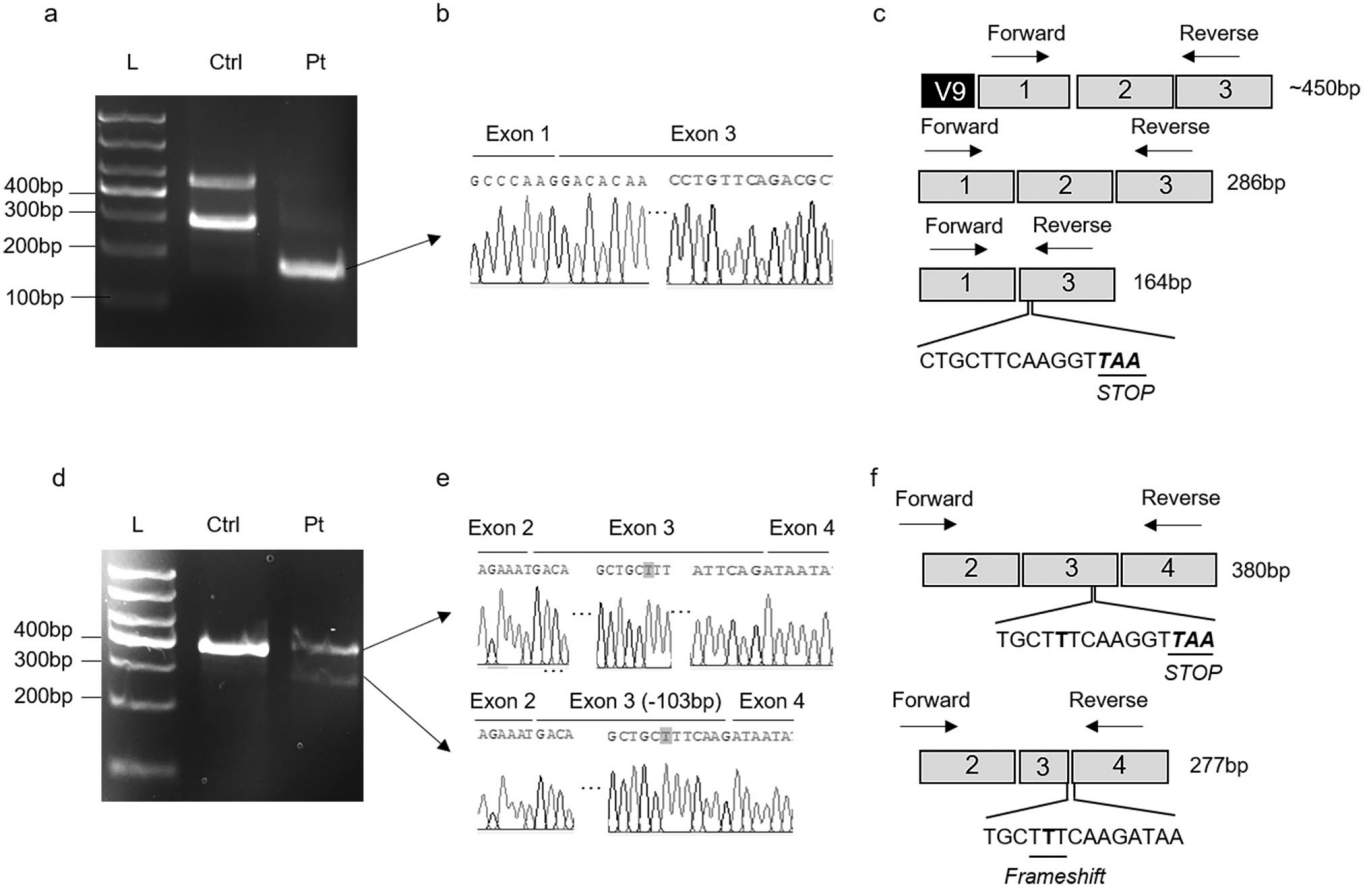
*TMEM126B* exons 2 and 3 splicing patterns in patient-derived lymphocytes. Agarose gel electrophoresis (**a**) and Sanger sequencing (**b**) for RT-PCR products from patient and healthy control’s lymphocytes, which the primers was designed at exon 1 and exon 3 respectively. **c** Schematic representation of the splicing process. The c.82-2 A > G mutation resulted in premature termination at position of 58 in exon 3. V9 referred to the transcript variant 9 of *TMEM126B* (NM_001350396.2). Agarose gel electrophoresis (**d**) and Sanger sequencing (**e**) for RT-PCR products from patient and healthy control’s lymphocytes, which the primers were designed at the junction of exon 1 and 2 and exon 4 respectively to avoid amplifying additional transcripts. The mutation was highlight with gray. **f** Schematic representation of the splicing process. The c.290dupT caused a frameshift at position 101 of the “T” duplication transcript and frameshift at the 103-deletion transcript

To assess the physiologic relevance of the splicing defects revealed by the minigene assay, we analyzed the splicing pattern of *TMEM126B* in patient-derived lymphocytes. RT-PCR using primers specific to exons 1 and 3 spanning the variant c.82-2 A > G generated a PCR product with complete exon 2 skipping in patient-derived sample (Fig. 4a, b), resulting in frameshift from codon 28 and premature termination at position 58 in exon 3 (Fig. 4c), thus leading to nonsense-mediated mRNA decay. Agarose gel analysis and Sanger sequencing of the RT-PCR products generated from patient-derived mRNA using primers specific to exons 2 and 4 detected a full-length transcript carrying the c.290dupT in exon 3 and a shorter mRNA with the c.290dupT and 103-bp deletion of exon 3 (Fig. 4d, e). Both of the above transcripts were predicted to cause frameshift (Fig. 4f), which would likely lead to transcript elimination via the nonsense-mediated decay pathway. Whereas a very low abundance transcript lacking 103-bp of exon 3 were detected in control lymphocytes, which corresponded to the transcript variant 9 of *TMEM126B* (NM_001350396.2) by sequence comparing. These results obtained from patient-derived RNA samples further confirmed that c.82-2 A > G and c.290dupT mutations induced splicing defects.

### Mitochondrial complex I content and assembly defect and mitochondrial dysfunction in patient-derived lymphocytes

**Fig. 5:**
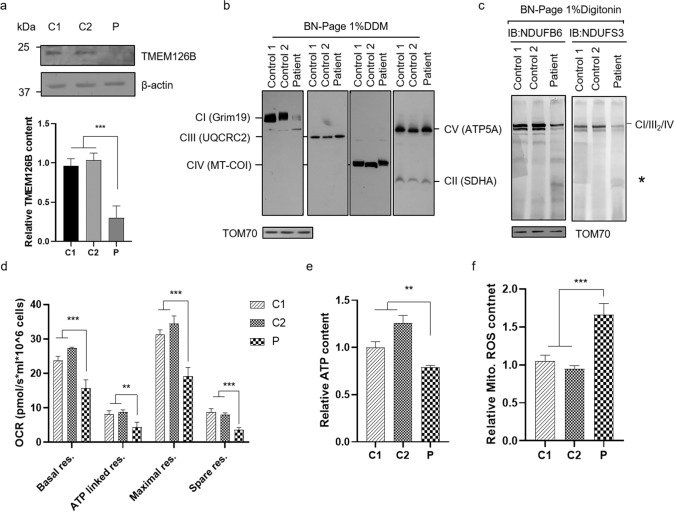
*TMEM126B* expression and mitochondrial functional validation in patient-derived lymphocytes. **a** Representative immunoblot and quantification analysis of *TMEM126B* protein levels in immortalized lymphocytes. BN-PAGE for mitochondrial complexes (**b**) and supercomplex (**c**). An asterisk indicates the incomplete assembly intermediate complex. TOM70 was used as internal loading controls. Oxygen respiration rate (**d**), relative cellular ATP (**e**) and mitochondrial ROS content (**f**) of healthy control and patient-derived lymphocytes. *p < 0.05, **p < 0.01, ***p < 0.001

To validate the pathogenic role of c.82-2 A > G and c.290dupT variants in *TMEM126B*, we first measured the protein expression level in patient-derived lymphocytes. As shown (Fig. 5a), compared with age-matched control, *TMEM126B* protein level was decreased by ~70% in patient-derived lymphocytes, which indicated the two variants dramatically resulted in *TMEM126B* deficiency. *TMEM126B* mainly participated in mitochondrial complex I assembly, thus OXPHOS supercomplexes and complexes were tested in patient-derived lymphocytes. The results indicated that the content of complex I was markedly decreased in patient-derived immortalized lymphocytes compared with normal controls. Moreover, supercomplex CI/III_2_/IV assembly was blocked, while lower assembly intermediate appeared to accumulate notably (Fig. 5b, c). Mitochondrial respiratory chain complexes are involved in maintaining proper mitochondrial function, and we then investigated mitochondrial functions in patient-derived immortalized lymphocytes. As shown in Fig. 5d, patient-derived lymphocytes showed a general decrease in cellular respiratory capacity, including basal, ATP-linked respiration, maximal respiration, and spare respiration capacity compared to controls. Cellular ATP content of patient-derived lymphocytes was significantly decreased, whereas the mitochondrial ROS level was increased (Fig. 5e, f). Together, these results demonstrated that mitochondrial OXPHOS function was severely impaired in patient‐derived lymphocytes carrying mutations of c.82-2 A > G and c.290dupT.

## Discussion

In this study, we firstly described two novel heterozygous mutations of *TMEM126B* (c.82-2 A > G and c.290dupT) from a Chinese patient clinically manifested with LLS. Both mutations resulted in frameshifts and premature termination. Protein expression indicated the two variants resulted in dramatically TMEM216B deficiency in patient-derived lymphocytes. Functional studies showed the impaired mitochondrial function in patient-derived immortalized lymphocytes due to severe complex I content and assembly defect. To the best of our knowledge, this is the first report of LLS in patient carrying mutations in *TMEM126B*.

TMEM126B was identified as the part of MCIA complex to co-migrate with other MCIA complex components (NDUFAF1, ECSIT, and ACAD9) by complexome profiling [10]. The defect of each component can cause heterogeneous clinical phenotypes. For example, mutations in *NDUFAF1* are mostly associated with cardiological symptoms [28, 29], including Wolff-Parkinson-White syndrome and hypertrophic cardiomyopathy. Genetically deficit in *ACAD9* commonly linked to cardiac symptoms, neurological symptoms, and severe lactic acidosis [30, 31]. Mendelian mutations in *ECSIT* gene has not yet reported. To better understand the genotype and phenotype of *TMEM216B*-related disorder, we summarized the published literature. As shown (Supplementary Table 2), individuals carrying *TMEM126B* mutations mainly presented with exercise intolerance, muscle weakness, hyperlactic acidemia, pure myopathy, chronic renal failure and cardiomyopathy [11–13]. Notably, the clinical phenotypes of our patient are consistent with those of patients carrying *TMEM126B* mutations previously reported, except for chronic renal failure and cardiomyopathy. Significantly, our patient show a more severe neurological symptoms with clinical presentation consistent with LLS, thus, we believe that patients with *TMEM126B* mutations may exhibit high clinical heterogenicity. The combination of clinical and molecular diagnosis are required for the diagnosis of *TMEM126B* mutation‐related mitochondrial diseases.

In silico analysis and in vitro experiments indicated that the c.82-2 A > G mutation located 2 bp before exon 2 could lead to the loss of the original 3’ splice acceptor site and the c.290dupT mutation located exon 3 might create a new constitutive splice acceptor site, which led to complete exon 2 skipping and an increase of transcripts with partial and complete deletion of *TMEM126B* exon 3, respectively. Exons contain sequence elements known as exonic splicing enhancers (ESEs), which function as binding sites for members of the serine/arginine-rich (SR) family of splicing factors and thus accurate pre-mRNA splicing [33, 34]. In addition, the ESEs/SR protein complexes play a role in ensuring that 5ʹ and 3ʹ splice sites within the same intron are used, thus suppressing exon skipping [32, 33]. Previous studies have shown that mutations located in ESE region could prevent the binding of SR proteins to ESE, which induced exon skipping [34]. Results of ESE finder software in this study suggested that c.82-2 A > G mutation barely impact the binding of SRP40, however, c.290dupT mutation may disrupt a putative SRp55 binding site, thus increasing the proportion of abnormal splicing transcripts.

Mitochondrial functional study indicated that patient-derived immortalized lymphocytes carrying biallelic mutations exhibited a global mitochondrial dysfunction with decreased mitochondrial respiratory capacity, reduced ATP content and increased mitochondrial ROS levels due to the complex I content and assembly defect. Unlike previous reports, biallelic mutations in our patient caused the complete deletion of exon 2 and the partial truncation of exon 3 of *TMEM126B*, which resulted in a more severe *TMEM126B* defect, thus leading to a more severe complex I deficiency and brain phenotypes.

In summary, we identify *TMEM126B* as a novel disease-causing gene associated with LLS, and report two novel *TMEM126B* mutations (c.82-2 A > G and c.290dupT) that cause splicing defects and lead to mitochondrial dysfunction due to the severe complex I deficiency. Our study not only expands the genetic mutation spectrum of LLS but the clinical spectrum caused by *TMEM126B* mutations, thus contributing to the clinical diagnosis of *TMEM126B* mutation-related mitochondrial diseases.

## Supplementary information

Supplement table 1

Supplement table 2
