# Supplementary material for: Novel biallelic mutations in TMEM126B cause splicing defects and lead to Leigh-like syndrome with severe complex I deficiency
Source: J Hum Genet. 2022 Dec 8;68(4):239–46. doi: 10.1038/s10038-022-01102-4 (PMC10040336; doi:10.1038/s10038-022-01102-4)
Supplement: Supplementary file 1 — Supplement table 1 [file 10038_2022_1102_MOESM1_ESM.pdf]

Supplement table 1 Summary of primers sequence

Primers	Sequence (5’→3’)	Purpose
bDupT-Fwd	TTTGTTTACTGTCCTATCTTAAGCAC	Variants validation primers for Sanger sequence from the candidate and her family members’ blood sample
bDupT-Rev	ACCTGATTAGGAGGATTGTTGC	
b82-2-Fwd	AAGCCAATGTCTAACAGATAAT	
b82-2-Rev	CTTGTTTCCCCCATTCTT	
pDupT-Fwd	CGCTCGAGTTTGTTTACTGTCCTATCTTAAGCAC	Minigene construction primers for the two mutation sites
pDupT-Rev	CGGGATCCACCTGATTAGGAGGATTGTTGC	
p82-2-Fwd	CGCTCGAGAA GCCAATGTCTAACAGATAAT	
p82-2-Rev	CGGGATCCCTTGTTTCCCCCATTCTT	
lymEx1-3- Fwd	ATGGTGGTGTTCGGGTATGA	RNA analysis primers for patient-derived lymphocytes
lymEx1-3- Rev	AGCGTCTGAACAGGAAGTTTG	
lymEx2-4-Fwd	GAAGCGCCCAAGGTTTTCAA	
lymEx2-4-Rev	ACTATGCCAATCAGTGAGCTTCT	
dSD-Fwd	TCTGAGTCACCTGGACAACC	RNA analysis primers for HEK293T cell lines
dSA-Rev	ATCTCAGTGGTATTTGTGAGC	
