# Supplementary material for: Novel biallelic mutations in TMEM126B cause splicing defects and lead to Leigh-like syndrome with severe complex I deficiency
Source: J Hum Genet. 2022 Dec 8;68(4):239–46. doi: 10.1038/s10038-022-01102-4 (PMC10040336; doi:10.1038/s10038-022-01102-4)
Supplement: Supplementary file 2 — Supplement table 2 [file 10038_2022_1102_MOESM2_ESM.pdf]

Supplement table 2 Summary of clinical and biochemical features of TMEM126B variants

Patient ID	P1	P2	P3	P4	P5
Gender	Female	Male	Male	Male	Male
Ethnicity	Chinses	American	Belgium	Belgium	Belgium
Age of onset	3.2 years	8 years	12 years	10 years	8 years
Clinical course	Alive	Alive	Wheelchair bound	Wheelchair bound	Alive
Mutation#1	c.82-2A>G	c.635G>T	c.401delA	c.401delA	c.401delA
Mutation#2	c.290dupT	c.635G>T	c.635G>T	c.635G>T	c.635G>T
Motor development delay	NA	NA	-	-	-
Exercise intolerance	NA	+	+	+	+
Muscle weakness	+	-	+	+	-
Retinitis pigmentosa	-	NA	+	-	-
Eye strabismus	+	NA	-	-	-
Fatigue	NA	+	-	-	+
Multisystem presentation	-	NA	-	-	-
Metabolic investigations	Blood lac↑	Blood lac↑	Blood and CSF lac↑	Blood lac↑	-
Neurological examination	Abnormal in bilateral cephalopods and pons	-	NA	NA	-
RC enzymes activity (*, muscle; **, fibroblasts)	NA	CI↓*; CII, CIII, CIV and CS↑*	CI↓*;CS↑*	CI↓*; CII and CS↑*	CI↓*; CII, CIII and CS↑*

RC, respirational complex; NA, not available; -, negative outcome; +, positive outcome. P1, this article, P2-P7 (11).

Supplement table 2 (continued)

Patient ID	P6	P7	P8	P9	P10	P11
Gender	Female	Female	Male	Female	Male	Female
Ethnicity	Belgium	Poland	European	European	European	Dutch
Age of onset	15 years	2 months	38 years	9 years	3 years	Birth
Clinical course	Alive	Alive	Alive	Alive	Alive	Alive
Mutation#1	c.401delA	c. 635G>T	c. 635G>T	c. 635G>T	c. 635G>T	c. 635G>T
Mutation#2	c.635G>T	c.635G>T	c. 397G>A	c. 208C>T	c.397 G>A	c. 635G>T
Motor development delay	-	-	-	-	-	+
Exercise intolerance	+	-	+	+	+	+
Muscle weakness	-	-	-	+	+	+
Retinitis pigmentosa	-	-	NA	-	-	-
Eye strabismus	-	-	NA	NA	-	-
Fatigue	+	-	+	NA	-	-
Multisystem presentation	-	+	-	NA	-	-
Metabolic investigations	-	Serum lac↑	Blood lac and alanine↑	Urine lac, blood lac and alanine↑	Urine lac, blood lac and alanine↑	Blood lac↑
Neurological examination	-	NA	-	NA	-	-
RC enzymes activity (*, muscle; **, fibroblasts)	CI↓*; CII, CIV and CS↑*	CI↓*; CS↑*	CI↓*†	CI↓*; III, CIV and CV↑* CII↑†, CIV and CV↓†	CI↓*, CIII and CIV↑†	CI↓*; CIV and CV↑*

RC, respirational complex; NA, not available; -, negative outcome; +, positive outcome. P8-P10 (12), P11 (13).
